# Genotype I African swine fever viruses emerged in domestic pigs in China and caused chronic infection

**DOI:** 10.1080/22221751.2021.1999779

**Published:** 2021-11-22

**Authors:** Encheng Sun, Lianyu Huang, Xianfeng Zhang, Jiwen Zhang, Dongdong Shen, Zhenjiang Zhang, Zilong Wang, Hong Huo, Wenqing Wang, Haoyue Huangfu, Wan Wang, Fang Li, Renqiang Liu, Jianhong Sun, Zhijun Tian, Wei Xia, Yuntao Guan, Xijun He, Yuanmao Zhu, Dongming Zhao, Zhigao Bu

**Affiliations:** State Key Laboratory of Veterinary Biotechnology, National High Containment Facilities for Animal Diseases Control and Prevention, National African Swine Fever Para-reference Laboratory, Harbin Veterinary Research Institute, Chinese Academy of Agricultural Sciences, Harbin, People’s Republic of China

**Keywords:** African swine fever virus, genotype I, virulence, pig, China

## Abstract

The Georgia-07-like genotype II African swine fever virus (ASFV) with high virulence has been prevalent in China since 2018. Here, we report that genotype I ASFVs have now also emerged in China. Two non-haemadsorbing genotype I ASFVs, HeN/ZZ-P1/21 and SD/DY-I/21, were isolated from pig farms in Henan and Shandong province, respectively. Phylogenetic analysis of the whole genome sequences suggested that both isolates share high similarity with NH/P68 and OURT88/3, two genotype I ASFVs isolated in Portugal in the last century. Animal challenge testing revealed that SD/DY-I/21 shows low virulence and efficient transmissibility in pigs, and causes mild onset of infection and chronic disease. SD/DY-I/21 was found to cause necrotic skin lesions and joint swelling. The emergence of genotype I ASFVs will present more problems and challenges for the control and prevention of African swine fever in China.

## Introduction

African swine fever (ASF) is a highly contagious, haemorrhagic swine disease caused by the African swine fever virus (ASFV) [1,2]. The disease continues to pose a major threat to the pig industry, food security, and rural development worldwide, and is listed as a notifiable disease by the World Organization for Animal Health (OIE).

The disease signs and mortality rates of ASF vary among viral strains and animal species [1]. Acute disease presents with high fever, depression, haemorrhages, cyanosis, and death within 15 days with near 100% mortality [3]. Subacute and chronic diseases are usually caused by less virulent strains with a longer course of disease and lower mortality. Chronic disease signs include intermittent fever, weight loss, chronic skin ulcers, and arthritis [1].

ASF was first described in Kenya in 1921 [1] and spread throughout sub-Saharan Africa (www.ioe.int). ASFVs are classified into different genotypes based on the 3′-end sequences of the B646L gene, which encodes the major capsid protein p72 [3,4]. To date, 24 different ASFV genotypes have been identified in Africa. Multiple virus strains and genotypes may circulate in any given region of Sub-Saharan Africa [3–5]. Genotype I virus was first found in Portugal outside Africa in 1957 and caused acute onset of infection [6]; it then appeared in Spain, France, Madeira, Italy, Cuba, Malta, Sardinia, Brazil, the Dominican Republic, and Haiti in the 1960s and 1970s [7–9]. Genotype I ASFVs have since been eradicated in all of these countries except Italy-Sardinia, where it has been endemic since 1978 [8].

In 2007, genotype II ASFV was first introduced into Georgia outside Africa and then spread to other Caucasian and European countries [6]. In 2018, Georgia-07-like genotype II ASFV emerged in China, and spread to 15 other Asian countries (www.oie.int) [10–12]. This virus causes very acute disease with near 100% mortality, and has been prevalent in China for almost three years (www.oie.int) [4,10,12–14]. A subsequent surveillance study found that lower virulent genotype II ASFVs emerged in China in 2020 due to natural mutations in the genomes of the highly virulent viruses [15]. These natural mutants showed lower virulence and high transmissibility, caused chronic and persistent infections in pigs, but were continuously shed via the oral and rectal routes at a low level, which causes more difficulties and challenges for the early diagnosis and control of ASF in China [15].

In the present study, we isolated genotype I ASFVs from pigs in two provinces in China and sequenced the whole viral genomes of two isolates for phylogenetic analysis. Specific-pathogen-free (SPF) pigs were inoculated with one of the viruses at different doses to determine its pathogenicity. Disease signs, lesions, viremia, contact transmission, and viral loads in different organs and tissues were assessed.

## Materials and methods

### Facility and ethics statements

All experiments including animal studies with live ASFVs were performed in the enhanced biosafety level 3 (P3+) facilities in the Harbin Veterinary Research Institute (HVRI) of the Chinese Academy of Agricultural Sciences (CAAS), which are approved for such use by the Ministry of Agriculture and Rural Affairs and the Animal Experimentation and Laboratory Animal Welfare Committee of HVRI.

### Cell culture and virus isolation

Primary porcine alveolar macrophages (PAMs) were obtained from bronchoalveolar lavage of SPF pigs as previously described [16]. Peripheral blood mononuclear cells (PBMCs) were prepared from EDTA-treated swine blood by using a Pig PBMC Isolation Kit (TBD sciences, China).

Homogenized pig tissue samples (ASFV p72 gene positive by qPCR) were used to inoculate primary PAMs. At three days post-inoculation (p.i.), the ASFVs in the cell supernatants and PAMs were detected by using qPCR. The cell supernatants were collected and used to inoculate PAMs to propagate virus stocks. Each virus stock was examined to confirm the lack of bacterial contamination, classical swine fever virus (CSFV), porcine respiratory and reproductive syndrome virus (PRRSV), pseudorabies virus (PRV), and porcine circovirus type 2 (PCV2). Virus stocks were aliquoted and stored at −80°C.

### HAD assay

PBMCs were cultured in 96-well plates and infected with 10-fold diluted ASFVs [10]. Haemadsorption in the cultures was observed for at least seven days by microscopy. The 50% HAD dose (HAD_50_) was measured by using the Reed and Muench method [17].

### Immunofluorescence assay

PAMs were seeded in 96-well plates and infected with different doses of ASFVs. Virus replication was confirmed by using an Immunofluorescence assay (IFA) with an ASFV-specific antibody, as described previously [15,18]. The 50% tissue culture infective dose (TCID_50_) was measured by using the Reed and Muench method [17].

### Electron microscopy

PAMs were cultured in 6-well plates and infected with the indicated ASFVs at an MOI of 0.2. At 48 h p.i., ASFV-infected cells were collected for morphological observation under an electron microscopy, as described previously [10].

### Quantitative PCR

ASFV genomic DNA from cell supernatants, whole peripheral blood samples, swabs, and tissue homogenates was extracted by using QIAamp® DNA Mini Kits (Qiagen, Germany). Quantitative PCR (qPCR) was carried out on an *ABI* QuantStudio5 (Q5) (ABI, USA) as previously described [19].

### Viral gene sequencing and genetic analysis

Viral genes and genome segments were amplified by PCR and sequenced by using the first generation Sanger DNA sequencing method [20]. Multiple-sequence alignments and phylogenetic analyses of viral genes and genome segments were carried out by using the software DNAStar and MEGA X. Primer sequences for PCR amplification and sequencing of genotype I ASFV genomes are available upon request. The genomes of the ASFVs were aligned by using E-INS-i of the program MAFFT v7 [21] and ambiguously aligned regions were excluded by using Gblocks-0.91 [22]. Phylogenetic analysis was hypothesized using maximum likelihood (ML), and ModelFinder was used to select the best-fit model according to the Bayesian information criterion (BIC) [23]. ML analysis was hypothesized using IQ-Tree [24] and the best model was TVM + F. Bootstrap branch support values (MLBS) were obtained with 1000 rapid bootstrap inferences and subsequently sought in a thorough ML search of the dataset.

### Animal experiments

SPF Large White and Land race-crossed pigs without PRRSV, PCV2, PRV, or CSFV infection were obtained from the Laboratory Animal Center of HVRI. The 7-week-old SPF pigs were divided into groups of six pigs, and then intramuscularly inoculated with different doses of ASFV. Two additional naïve pigs were cohoused with the infected pigs from the first day of challenge to determine ASFV contact transmission. Each pig was monitored daily for body temperature changes and disease signs including anorexia, depression, fever, purple skin discolouration, staggering gait, diarrhoea, and cough for 28 days p.i. or post-contact (p.c.). Oral and rectal swabs, and blood were collected for virus detection by using qPCR at different timepoints p.i. or p.c … Necropsy was immediately performed if an animal died. Tissue samples including heart, liver, spleen, lung, kidney, tonsil, thymus, adrenal gland, marrow, synovial fluid, and lymph nodes (intestinal lymph node, inguinal lymph node, submaxillary lymph node, bronchial lymph node, gastrohepatic lymph node, and mediastinal lymph node) were collected during necropsy, and viral DNA was detected by using qPCR. Serum samples were collected and assessed for IgG against the ASFV p72 protein by using a commercial ELISA kit (Harbin Weike Biotechnology Co., Ltd, China).

## Results

### Isolation of genotype I ASFVs in China

In June 2021, a fattening pig weighing about 80 kg showed paralytic symptoms on a farm in Shandong province, and was euthanized for autopsy. The lung sample was collected and delivered to the Chinese National African Swine Fever Para-reference Laboratory (CNASFPL) for ASFV detection. On another farm in Henan province, the fattening pigs developed chronic infection signs including weight loss, intermittent fever, skin ulcers, and arthritis; sporadic deaths were also observed. Samples from four dead pigs, including lymph nodes and spleens, were collected and delivered to the CNASFPL for ASFV detection. All samples were confirmed to be ASFV positive by qPCR targeting the viral p72 gene [19]. Further sequence analysis of the p72 genes indicated that the ASFVs in these samples belonged to genotype I.

**Figure 1.**
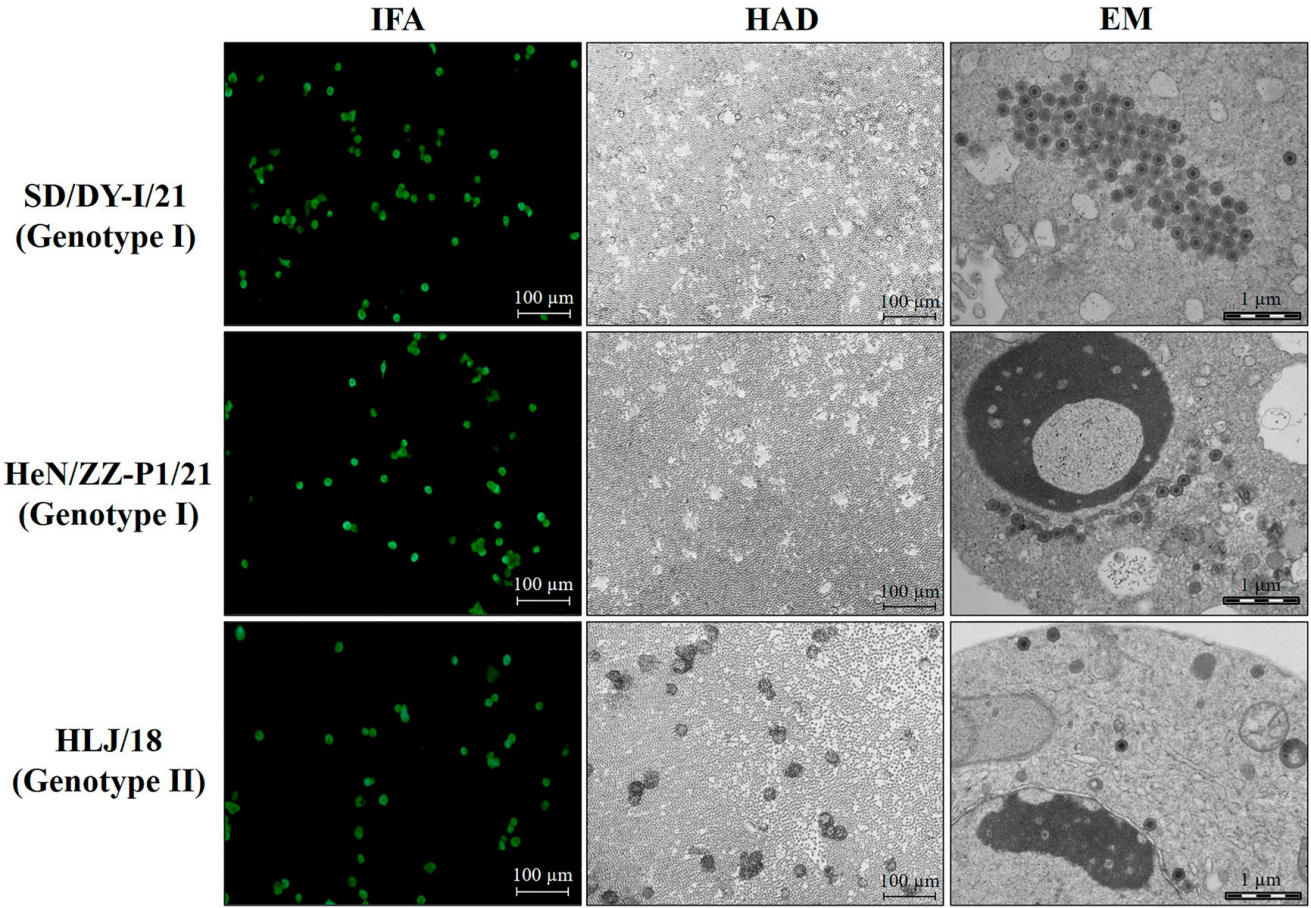
Characterization of genotype I ASFV isolates *in vitro*. PAMs were infected with the genotype I isolates SD/DY-I/21 and HeN/ZZ-P1/21, and the genotype II strain HLJ/18 as a control at an MOI of 0.1. The cells were fixed and analysed by using an immunofluorescence assay (IFA) at 24 h p.i. The haemadsorption (HAD) assay was performed with the indicated viruses in PBMCs. PAMs were infected with ASFVs in 6-well plates (MOI=0.2), and the cell pellets were harvested for morphological assessment by using an electron microscope (EM).

Subsequently, two genotype I ASFVs were isolated from the samples from the Shandong and Henan province farms, and were named Pig/Shandong/DY-I/2021 (SD/DY-I/21) and Pig/Henan/ZZ-P1/2021 (HeN/ZZ-P1/21), respectively. Both isolates replicated well in PAMs with viral titres of >5 × 10^7^ TCID_50_/ml in cell culture supernatants, and showed non-haemadsorption activity (Figure 1). Electron microscopy (EM) observation revealed typical morphology of ASFV particles within the infected PAMs (Figure 1).

### Genome analysis of the genotype I ASFV isolates SD/DY-I/21 and HeN/ZZ-P1/21

**Figure 2.**
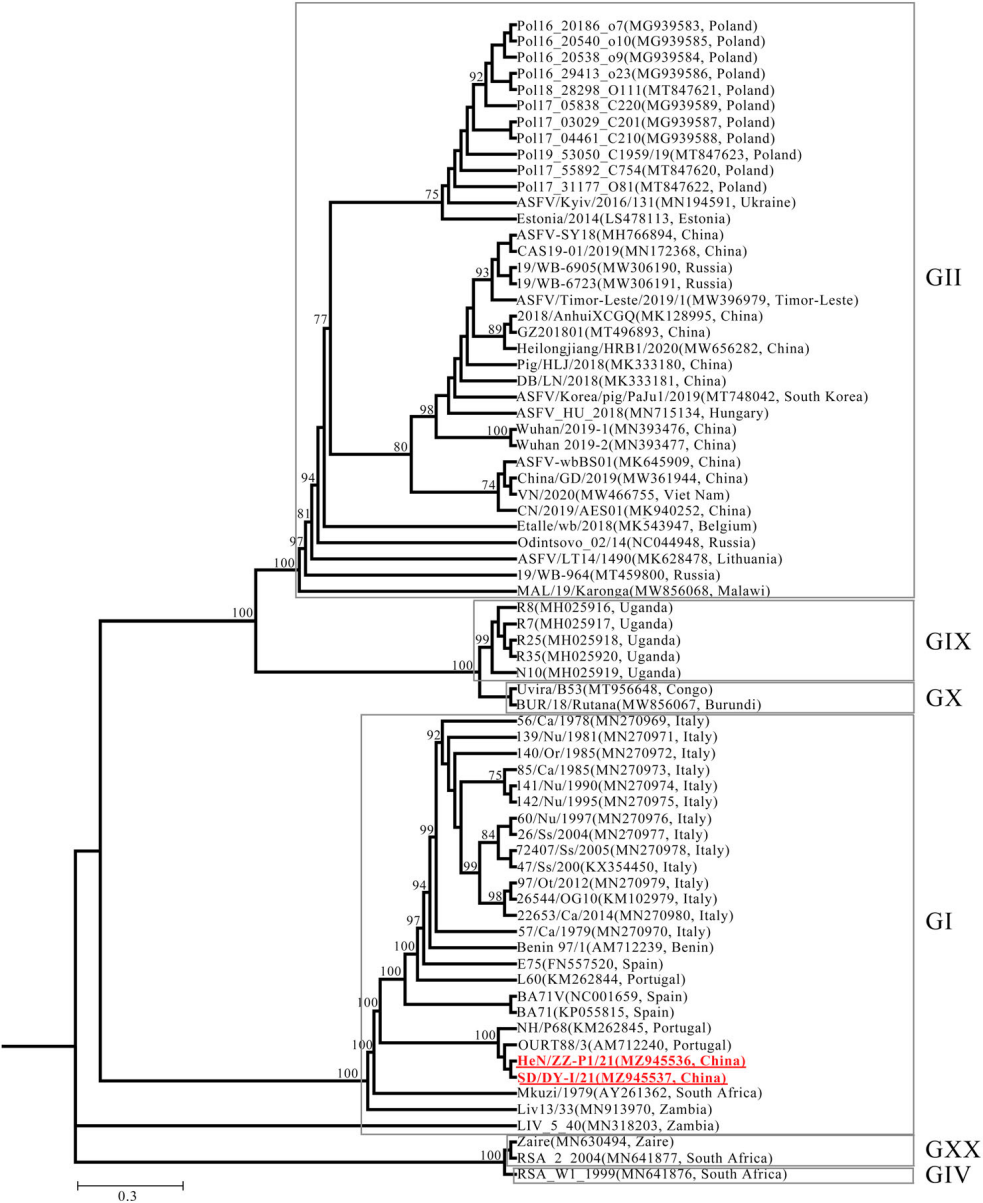
Phylogenetic tree based on the full genome sequences derived from SD/DY-I/21, HeN/ZZ-P1/21, and 70 reference strains from the GenBank database (accession numbers are reported in brackets). GI, all strains are genotype I ASFVs. GII, all strains are genotype II ASFVs. The red boldface type indicates the isolates in this study.

To characterize the phylogenic specificity of the genotype I ASFVs isolated in China, we sequenced the whole genomes of SD/DY-I/21 and HeN/ZZ-P1/21 by using a segmentation PCR strategy described previously [12]. The sequence data have been deposited in the GenBank and the accession numbers are MZ945537 and MZ945536. The phylogenetic tree was constructed by using the whole genome sequences of SD/DY-I/21 and HeN/ZZ-P1/21, as well as 70 reference strains in the GenBank database (Figure 2). SD/DY-I/21 and HeN/ZZ-P1/21 belong to the same clade with the genotype I Portuguese isolates NH/P68 (GenBank: KM262845) and OURT88/3 (GenBank: AM712240), and differ from the previous genotype II ASFV isolates in China (Figure 2). Sequence analysis showed 172, 025 bp and 158 ORFs for SD/DY-I/21 and 171, 235 bp and 157 ORFs for HeN/ZZ-P1/21.

**Figure 3.**
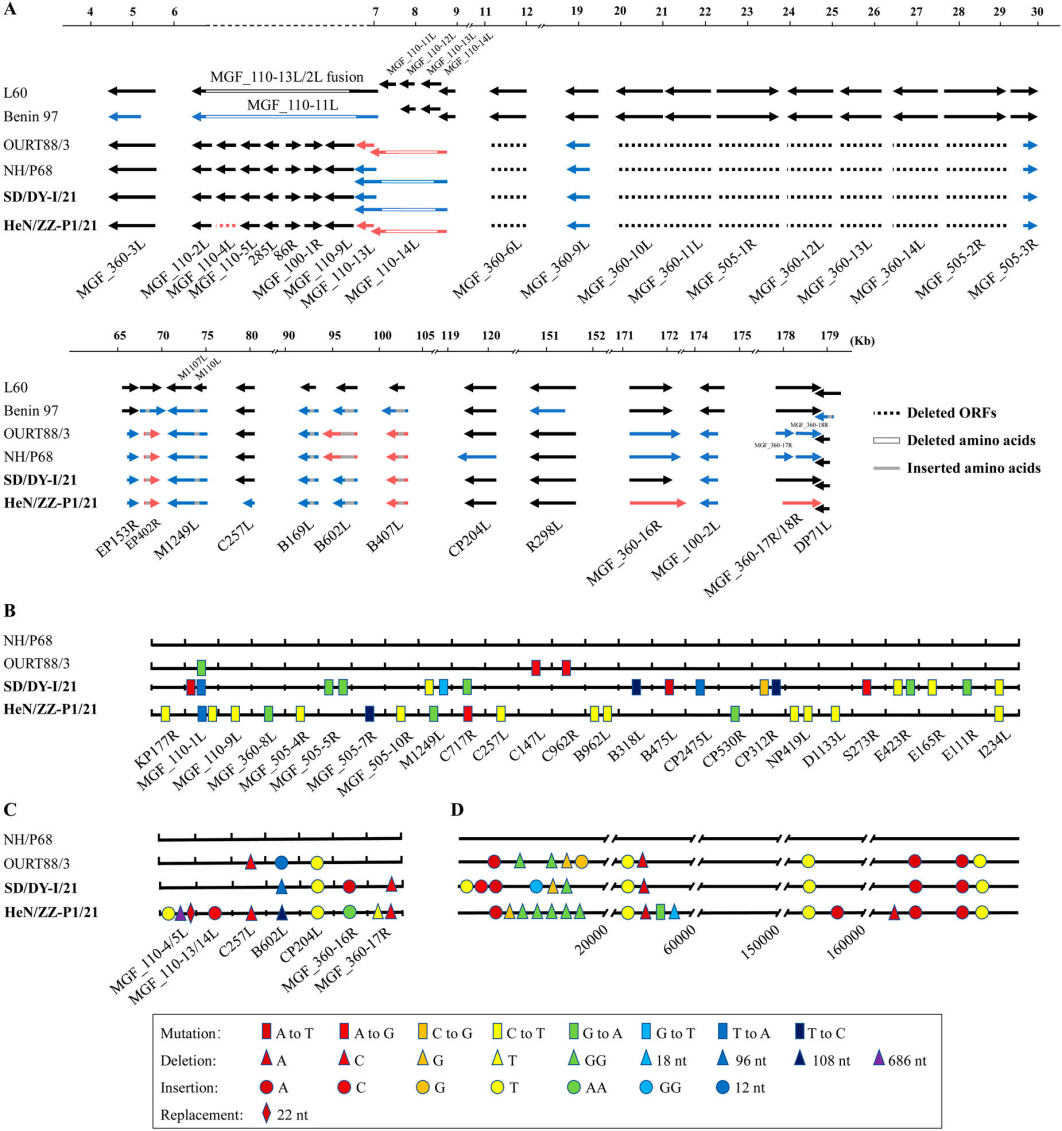
ORFs, nucleotide mutations, deletions, insertions, and replacement in the genomes of SD/DY-I/21 and HeN/ZZ-P1/21. Analysis of the deletion, shortening and lengthening of all ORFs of SD/DY-I/21 and HeN/ZZ-P1/21 compared with virulent isolates L60 and Benin 97, and attenuated isolates NH/P68 and OURT88/3 (A). The whole genome sequences of SD/DY-I/21 and HeN/ZZ-P1/21 were respectively compared with those of low virulence isolates NH/P68 and OURT88/3 for nucleotide mutations in ORFs (B), nucleotide deletions, insertions, and replacement in ORFs (C); and nucleotide mutations, deletions, and insertions in the noncoding regions (D). The names of the ORFs are shown on the bottom of each panel.

Compared to the whole genomes of virulent L60 and Benin 97 strains, SD/DY-I/21 and HeN/ZZ-P1/21 as well as NH/P68 and OURT88/3 deleted 10 ORFs (MGF_110-11L, MGF_110-12L, MGF_360-6L, MGF_360-10L, MGF_360-11L, MGF_505-1R, MGF_360-12L, MGF_360-13L, MGF_360-14L, and MGF_505-2R), and had 5 truncated ORFs (MGF_360-9L, MGF_505-3R, EP153R, EP402R, and MGF_100-2L) (Figure 3(A)). Phylogenetic analysis of B646L, E183L, and B602L genes grouped L60, Benin 97, NH/P68, OURT88/3, SD/DY-I/21, and HeN/ZZ-P1/21 to other genotype I ASFV strains from Europe and West Africa (Supplementary Figure S1), consistent with the previous assumption that all genotype I ASFVs were originally derived from Africa [25]. The central variable region (CVR) profiles of the B602L gene of SD/DY-I/21 and HeN/ZZ-P1/21 showed no similarity with that of publicly available ASFVs including NH/P68 and OURT88/3 (Supplementary Table S1).

Compared to the whole genome of NH/P68 in the coding regions, SD/DY-I/21 differs by 18 single nucleotide changes in 13 open reading frames (ORFs), resulting in 12 amino acid (AA) changes (Figure 3(B) and Supplementary Table S2); HeN/ZZ-P1/21 differs by 18 single nucleotide mutations in 15 ORFs, resulting in 13 AA changes (Figure 3(B) and Supplementary Table S2); and OURT88/3 has 3 single nucleotide changes in 3 ORFs (Figure 3(B)). SD/DY-I/21 has 96 nucleotides deleted from sites 87541 to 87636 in its B602L ORF, a single C deletion at site 169370 in its MGF_360-17R ORF, a single T insertion at site 110751 in its CP204L ORF, and a single A insertion at site 163390 in its MGF_360-16R ORF (Figure 3(C)); HeN/ZZ-P1/21 has a 686-nucleotide deletion from sites 7105 to 7790 in MGF_110-4/5L, a 22-nucleotide fragment replacement from sites 8123 to 8144 in MGF_110-5L, a single C deletion at site 11607 in MGF_110-13/14L, 108 nucleotides deleted from sites 87456 to 87563 in its B602L ORF, single T and C deletions at sites 169200 and 169370, respectively, in MGF_360-17R, a single C insertion at site 70851 in its C257L ORF, a single T insertion at site 110751 in its CP204L ORF, and two A insertions at site 163390 in its MGF_360-16R ORF (Figure 3(C)).

Compared to the whole genome of NH/P68 in the non-coding regions, SD/DY-I/21 has 4 nucleotides deleted at 3 positions and 10 nucleotides inserted at 9 positions (Figure 3(D)); HeN/ZZ-P1/21 has one mutation, 31 nucleotides deleted at 9 positions, and 7 nucleotides inserted at 7 positions (Figure 3(D)); OURT88/3 has 6 nucleotide deletions at 4 positions, and 7 nucleotides inserted at 7 positions (Figure 3(D)).

Compared to NH/P68, the nucleotide deletions and insertions in the genome of SD/DY-I/21 resulted in changes in 4 ORFs: three ORFs (MGF_360-16R, CP204L, and B602L) had deletions ranging from 2 to 32 AAs in length, and one ORF (MGF_360-17R) had an insertion of 182 AAs in length (Supplementary Table S2); the nucleotide deletions, insertions, and replacement in the genome of HeN/ZZ-P1/21 resulted in changes in 9 ORFs: one ORF (MGF_110-4L) exists in NH/P68 but not in HeN/ZZ-P1/21, one ORF (MGF_110-5L) has 4 AA mutations, 5 ORFs (MGF_110-13L, MGF_110-14L, B602L, CP204L, and C257L) have deletions ranging from 3 to 150 AAs in length, and two ORFs (MGF_360-16R and MGF_360-17R) have insertions of 41 and 165 AAs, respectively (Supplementary Table S2).

It is surprising that the HeN/ZZ-P1/21 genome differs significantly from that of SD/DY-I/21: 32 nucleotide mutations in 23 ORFs, 700 nucleotide deletions in 4 ORFs, 3 nucleotide insertions in 3 ORFs, and a replacement of 22 nucleotides in MGF_110-5L (Figure 3 and Supplementary Table S2). These results indicate that the two genotype I ASFV isolates, SD/DY-I/21 and HeN/ZZ-P1/21, come from different sources.

### The genotype I ASFV isolate SD/DY-I/21 causes chronic infection in pigs

**Figure 4.**
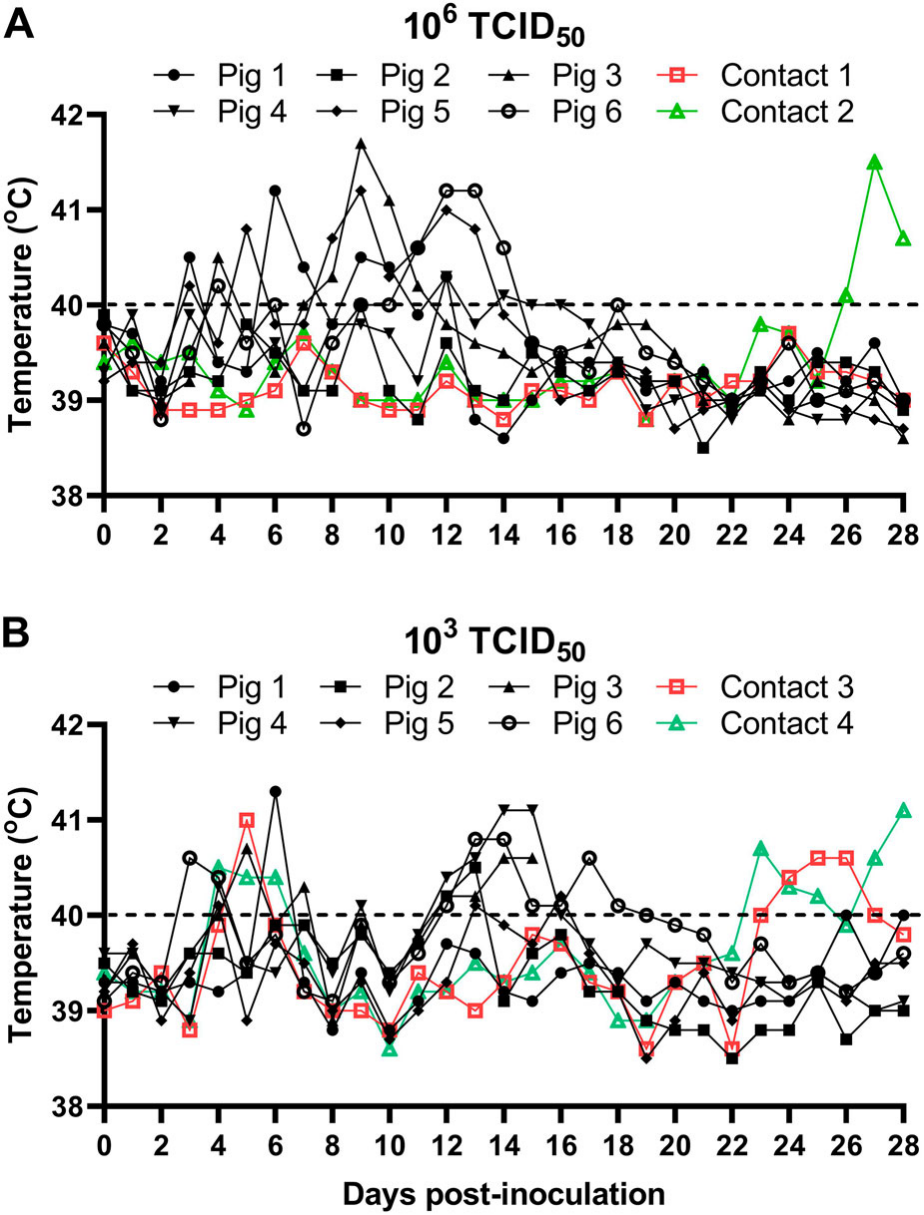
Rectal temperature changes in pigs infected with SD/DY-I/21 at a dose of 10^6^ TCID_50_ (A) or 10^3^ TCID_50_ (B). Pig 1–Pig 6, 6 pigs inoculated with SD/DY-I/21, Contact 1–Contact 4, 4 non-inoculated pigs cohoused to test for contact transmission. The dashed black lines in these panels indicate the threshold of normal rectal temperature.

**Figure 5.**
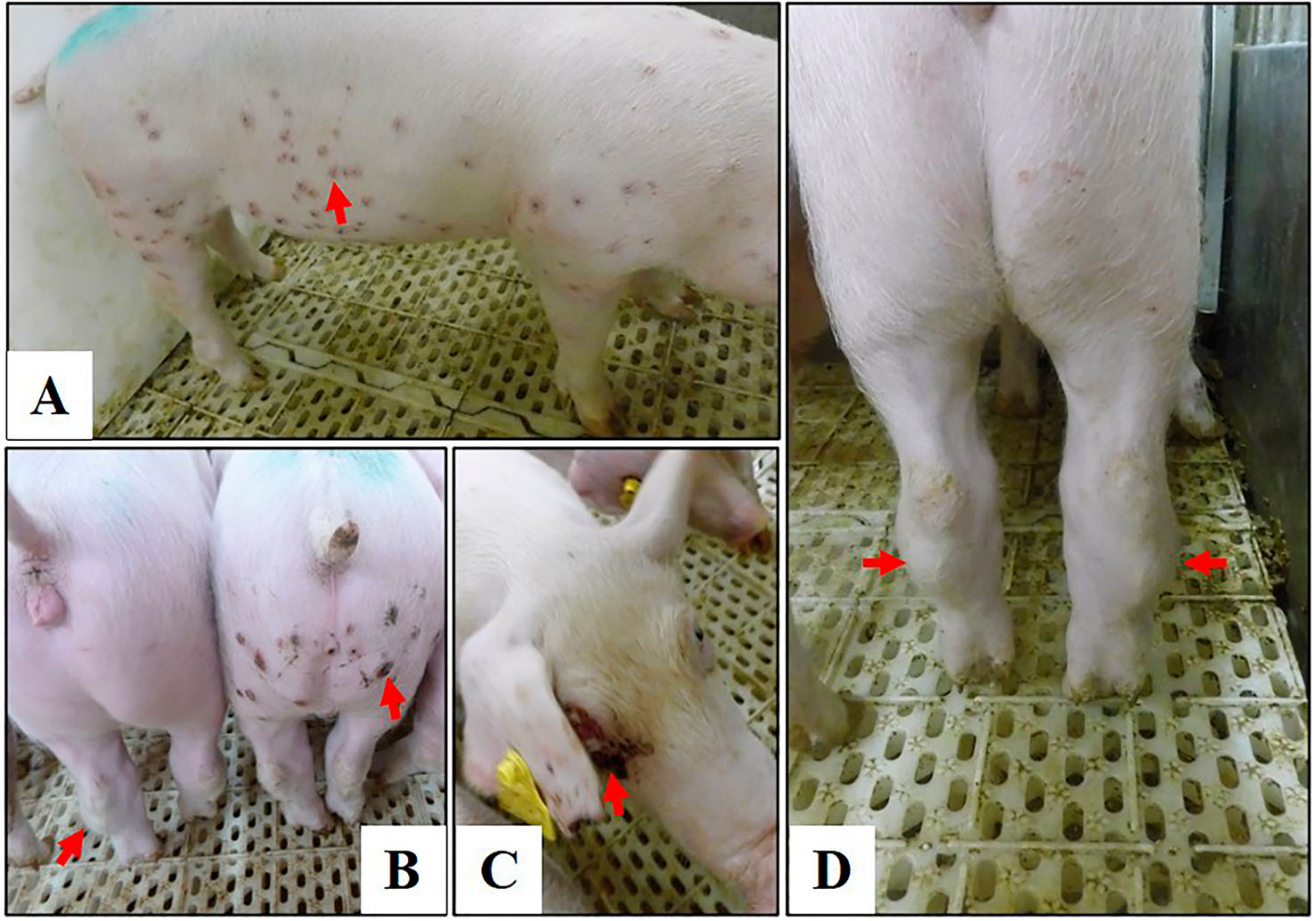
Disease signs in pigs infected with the genotype I isolate SD/DY-I/21. Disease signs include papules (A,B), cutaneous necrosis (B,C), and arthroncus of hind legs (B,D) in surviving pigs.

To determine the virulence of genotype I ASFVs isolated in China, SD/DY-I/21 was tested in pigs. Groups of six SPF pigs were intramuscularly inoculated with 10^3^ or 10^6^ TCID_50_ of SD/DY-I/21, respectively. All the challenged pigs showed intermittent fever of different degrees from Day 3 to 18 p.i. (Figure 4 and Table 1). In the 10^6^ TCID_50_-inoculated group, three pigs developed papules in the skin of neck, ear hind, abdomen, or even the whole body from Day 11 p.i. (Table 1 and Figure 5). All six pigs started to develop arthroncus from Days 13 and 17 p.i.; two pigs started to limp on Days 14 and 25 p.i., respectively (Table 1 and Figure 5). Two pigs developed multiple focal cutaneous necrosis on Days 17 and 20 p.i., respectively (Table 1 and Figure 5). All of the pigs survived for the duration of the 28-day observation period (Table 1 and Figure 5).

**Table 1.** Disease signs in pigs inoculated with different doses of the genotype I ASFV SD/DY-I/21.

Group	Treatment	Pig No.	Earliest appearance of disease signs (Day post-inoculation, dpi)						
			Fever (>40 ^o^C)	Papule	Arthroncus	Limp	Phyma	Cutaneous necrosis	Death
10^6^ TCID_50_	Infection	1	3	13	14	/ ^a^	/	/	NA^b^
		2	9	/	14	/	/	/	NA
		3	4	/	13	25	/	17	NA
		4	12	/	14	14	/	/	NA
		5	3	12	17	/	/	/	NA
		6	4	11	17	/	/	20	NA
	Contact	1	/	/	/	/	/	/	NA
		2	26	/	17	/	/	/	NA
10^3^ TCID_50_	Infection	1	6	13	14	/	/	/	NA
		2	12	13	14	/	/	/	NA
		3	4	13	14	/	/	/	16
		4	4	/	14	/	/	/	NA
		5	4	/	/	/	/	/	NA
		6	3	/	14	/	/	/	NA
	Contact	3	5	/	/	/	25	/	NA
		4	4	/	/	/	/	/	NA

^a^ No signs of disease.

^b^ The pig survived the infection.

In the 10^3^ TCID_50_-inoculated groups, three pigs developed sporadic papules in the skin from Day 13 p.i., and five pigs started to develop arthroncus from Day 14 p.i. (Table 1 and Figure 5). One pig developed disease and died on Day 16 p.i. (Table 1). Nostril bleeding before death, and hyperaemia and swelling of the spleen, liver, and lymph nodes were observed on autopsy. The remaining five pigs survived for the duration of the 28-day observation period (Table 1). These results demonstrate that the genotype I ASFV SD/DY-I/21 is pathogenic and mainly causes chronic disease signs in pigs.

### Replication and shedding of SD/DY-I/21 in pigs

**Figure 6.**
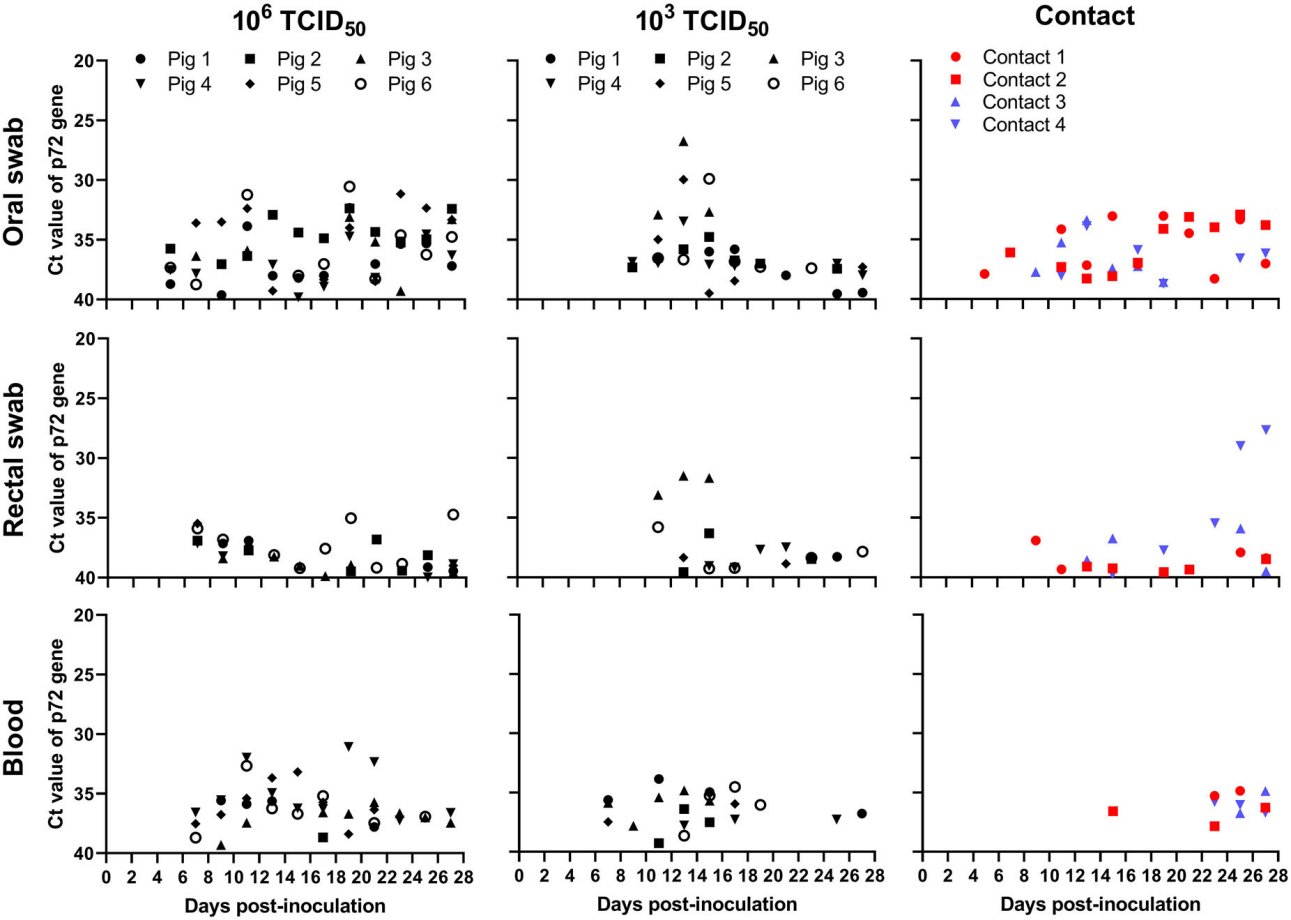
Detection of virus shedding and viremia in infected and contact pigs by use of qPCR. Oral and rectal swab samples, as well as blood, were collected from pigs infected with SD/DY-I/21 and contact pigs at the indicated days post-infection. Viral DNA was extracted and detected by using qPCR. The data on the contact pigs cohoused with the 10^6^ TCID_50_-inoculated pigs and 10^3^ TCID_50_-inoculated pigs are labelled in red and blue, respectively. The different shaped black dots represent individual pigs.

To evaluate viral shedding and viremia, oral and rectal swabs and blood samples from the pigs were collected to detect ASFV genome DNA by using qPCR every other day p.i.. For the pigs inoculated with 10^6^ TCID_50_ of SD/DY-I/21, viral DNA was detectable in the oral swabs from Day 5 p.i., in the rectal swabs from Day 7 p.i., and in the blood from Day 7 p.i. (Figure 6). For the pigs inoculated with 10^3^ TCID_50_ of virus, viral DNA was detectable in the oral swabs from Day 9 p.i., in the rectal swabs from Day 11 p.i., and in the blood from Day 7 p.i.. Viral DNA was detected in the oral and rectal swabs for more than 28 days after infection in all inoculated pigs (Figure 6). In general, virus shedding was detected earlier and at a higher level in the oral swabs than in the rectal swabs (Figure 6).

**Figure 7.**
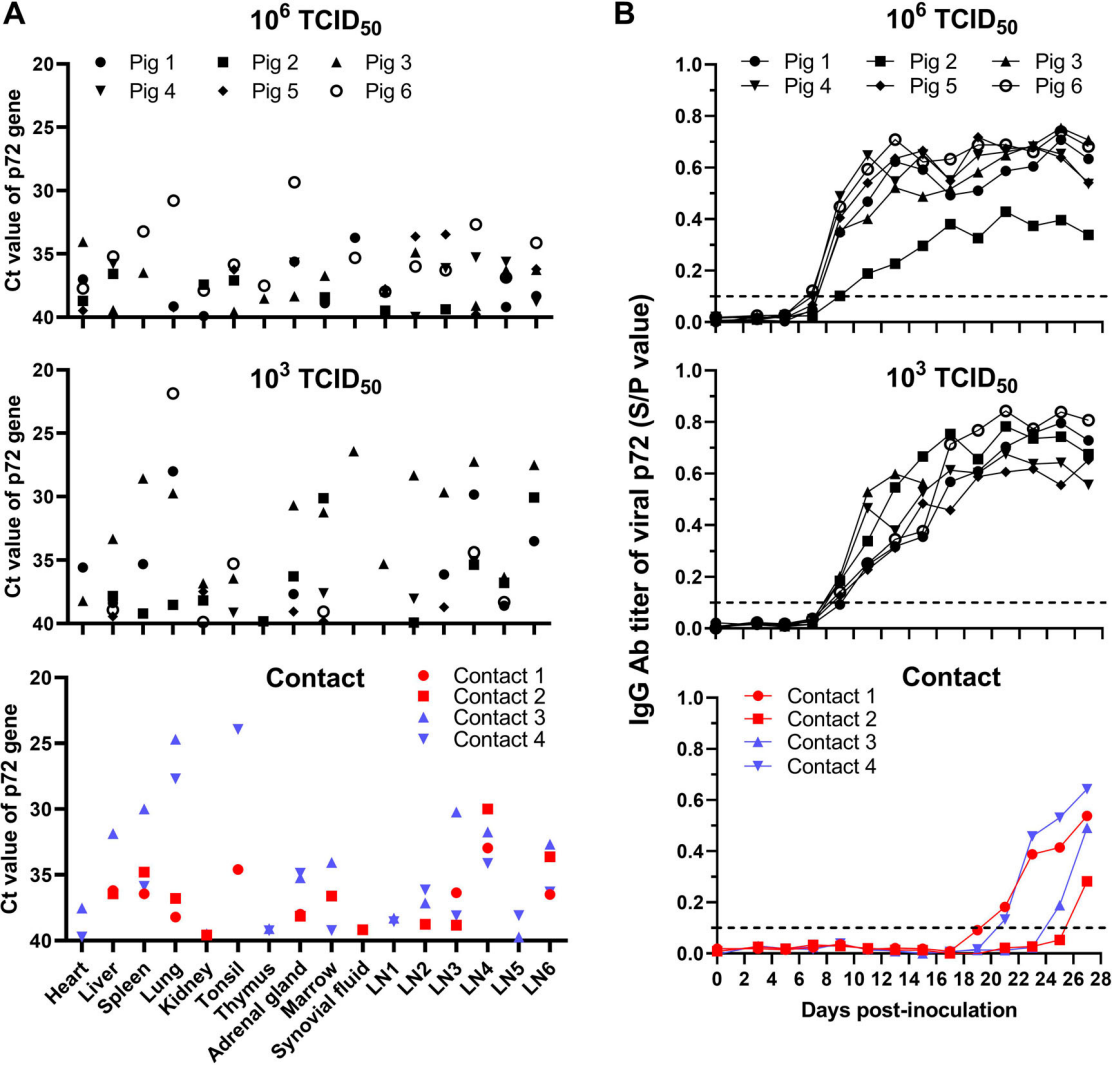
Detection of virus load in tissues and serum conversion in SD/DY-I/21-infected pigs and contact pigs. (A) The indicated tissue samples were collected from the dead pig and surviving pigs that were euthanized on Day 28 post-inoculation or post-contact to detect viral DNA by using qPCR. LN1, intestinal lymph node; LN2, inguinal lymph node; LN3, submaxillary lymph node; LN4, bronchial lymph node; LN5, gastrohepatic lymph node; and LN6, mediastinal lymph node. (B) ASFV-specific antibody in sera from infected and contacted pigs was detected at the indicated times post-infection or -contact by using a commercial ELISA kit coated with viral p72 protein. The data on the contact pigs cohoused with the 10^6^ TCID_50_- and 10^3^ TCID_50_-inoculated pigs are labelled in red and blue, respectively. The different shaped black dots represent individual pigs.

Tissue samples were collected from the one necropsied pig on the day of its death and from all of the surviving pigs, which were euthanized on Day 28 p.i., for viral genome DNA detection by qPCR. The viral loads in the tissues from the dead pig inoculated with the 10^3^ TCID_50_ dose were much higher than those of the surviving pigs (Figure 7(A)). Viral DNA was detected in the tissues of all of the surviving pigs, especially in the spleens, lungs, adrenal gland, marrow, and certain lymph nodes **(**Figure 7(A)).

### Transmission of genotype I ASFV SD/DY-I/21 in pigs

Two naive pigs were cohoused with pigs inoculated with 10^3^ TCID_50_ or 10^6^ TCID_50_ of SD/DY-I/21 from the first day of infection for contact transmission test, respectively. One contact pig in the 10^6^ TCID_50_ group started to have a fever from Day 26 p.c. (Figure 4(A) and Table 1) and developed arthroncus from Day 17 p.c. (Table 1). Viral DNA was detectable in oral swabs from Days 5 and 7 p.c., respectively, in the rectal swabs from Days 9 and 13 p.c., respectively, and in the blood from Days 15 and 23 p.c., respectively (Figure 6). Both contact pigs survived for the duration of the 28-day observation period, and were euthanized on Day 28 p.c.. Tissue samples were collected for viral DNA detection by qPCR. Low viral DNA loads were detected in different tissues of both contact pigs (Figure 7(A)).

Both contact pigs in the 10^3^ TCID_50_ group showed varying degrees of intermittent fever during the 28-day observation period (Figure 4(B)). One contact pig developed phyma on its ridge on Day 25 p.c. (Table 1). Viral DNA was detectable in the oral swabs from Days 9 and 11 p.c., respectively, in the rectal swabs from Days 13 and 15 p.c., respectively, and in the blood from Days 23 and 25 p.c., respectively (Figure 6). Both pigs survived for the duration of the 28-day observation period and were euthanized on Day 28 p.c., when tissues were collected for viral DNA detection by qPCR. High viral DNA loads were detected in the lungs of both contact pigs and in the tonsil of one contact pig (Figure 7(A)). Low viral DNA loads were detected in other tissues including heart, thymus, adrenal gland, marrow, and certain lymph nodes (Figure 7(A)).

### ASFV-specific antibody responses in inoculated and contact pigs

To evaluate the antibody response after SD/DY-I/21 infection, sera from inoculated and contact pigs were collected every other day p.i. or p.c. to detect IgG against ASFV p72 protein by using an ELISA. In the 10^6^ TCID_50_-inoculated group, antibody was detected in two pigs on Day 7 p.i., and in all six pigs from Day 9 p.i. (Figure 7(B)). In the 10^3^ TCID_50_-inoculated group, antibody was detected in five pigs on Day 9 p.i., and in all six pigs from Day 11 p.i. (Figure 7(B)). The antibody levels in pigs inoculated with virus gradually increased until Day 25 p.i. (Figure 7(B)). Two contact pigs in the 10^6^ TCID_50_ group seroconverted on Days 21 and 27 p.i., respectively, and two contact pigs in the 10^3^ TCID_50_ group seroconverted on Days 21 and 25 p.i., respectively (Figure 7(B)). These data further demonstrate that SD/DY-I/21 is highly transmissible in pigs.

## Discussion

In this study, we isolated and characterized what we believe are the first genotype I ASFV strains in China. Two genotype I isolates, SD/DY-I/21 and HeN/ZZ-P1/21, were isolated from domestic pig farms in two provinces. Phylogenetic analysis based on the whole genome sequences suggested that both viruses are highly similar to NH/P68 and OURT88/3, two genotype I Portuguese early isolates. NH/P68 was isolated from a domestic pig with chronic clinical signs in Portugal in 1968 [26–28], and OURT88/3 was isolated from soft ticks (*Ornithodoros erraticus*) on a Portuguese pig farm in 1988 [29]. However, HeN/ZZ-P1/21 and SD/DY-I/21 showed substantial genetic differences from NH/P68 and OURT88/3 in terms of nucleotide mutations, deletions, insertions, and short-fragment replacement across the whole genome. Of particular interest, whole genome sequence analysis clearly showed differences between HeN/ZZ-P1/21 and SD/DY-I/21. These results provide important information to help trace the source of these viruses through field surveillance and imply that two separate events may have led to the emergence of these genotype I ASFVs in China.

The genotype I Portuguese ASFV isolate was attenuated by serial passage in bone marrow cell cultures and then tested in the field in Portugal in the 1960s [30,31]. As chronic ASF developed post-vaccination, the field trial for this vaccine candidate was soon stopped [32]. After that, NH/P68 and OURT88/3 were isolated in the field [25,26,29], even though there is still no definitive proof for their origins. Through 30 years of efforts, Portugal and Spain successfully eradicated ASF in the 1990s (www.oie.int)[31]. NH/P68, OURT88/3, and similar viruses have not been detected in the field since the 1990s [2,31]. Lower virulent genotype II ASFVs can be evolved from their parental field isolates [15,33,34]. Genotype I ASFVs have been continuously circulating in Africa since 1921, and there is no doubt that attenuated genotype I viruses emerge in nature. However, the genomes and phenotypes of these isolates in Africa have been little characterized. It is one of the possibilities that the attenuated genotype I isolates including NH/P68 and OURT88/3 may be introduced from Africa. Therefore, how these genotype I viruses invaded China needs further investigation.

Animal challenge tests revealed that the genotype I ASFV isolated in China shows similar low pathogenicity in pigs to that of NH/P68 and OURT88/3. The SD/DY-I/21 isolate showed moderate virulence and efficient transmissibility in pigs and caused chronic disease and even death in one pig. Except for one contact pig, all of the pigs in our study developed fever. Moreover, almost all of the inoculated and contact pigs developed joint swelling. It has been reported that NH/P68 and OURT88/3 have low pathogenicity and cause chronic and persistent infection in domestic pigs [26,29]. NH/P68 infection caused 47% of pigs to experience clinical disease including necrotic skin areas and joint swelling, 79% of pigs had high fever, and 84% of pigs had viremia [26]. Less severe post-vaccination reactions including fever and joint swelling were apparent with OURT88/3 [29]. Mutations in the EP402R gene that encodes CD2v may cause the virus to lose its haemadsorbing activity and could partially attenuate its virulence in domestic pigs and wild boar [15,33–35]. MGF505/360 regions including MGF_505-1R, -2R, -3R, and MGF_360-12L, -13L, -14R genes are involved in virus replication in tick cells [36], the inhibition of interferon (IFN) production [37], and virus virulence in pigs [38]. Our genotype I isolates, HeN/ZZ-P1/21 and SD/DY-I/21, both had the truncated CD2v protein with non-haemadsorbing phenotype, and deleted MGF505/360 regions as same as NH/P68 and OURT/88, but different from virulent L60 and Benin 97 [25,39]. The change of these genes may partially explain the low virulence phenotype of these viruses.

Since 2018, Georgia-07-like genotype II ASFVs with high virulence have been prevalent in China. The naturally mutated genotype II low virulent strains were found in the field in 2020 [15]. Now, NH/P68- and OURT88/3-like genotype I epidemic strains with lower virulence than attenuated genotype II viruses have emerged in the field. These low virulent ASFVs have longer incubation periods, efficient transmissibility, and cause mild onset of infection and chronic disease. Infected pigs continuously shed viruses and develop low-level viraemia, which makes early diagnosis more difficult than attenuated genotype II viruses in the field. Meanwhile, new reassortants with unknown virulence among genotype II virulent and attenuated viruses, and genotype I viruses may subsequently emerge in the field. Therefore, the emergence of the genotype I ASFVs will cause more problems and pose bigger challenges for ASF eradication in China. The newly emerging genotype I ASFVs may cause severe and continuous economic losses to the pig industry, once they spread in swine herds or infect breeding sows and boars. Nationwide surveillance of genotype I ASFVs is therefore urgently needed to minimize the losses caused by their infection in China.
